# Identification and Optimization of Contributing Factors for Precocious Puberty by Machine/Deep Learning Methods in Chinese Girls

**DOI:** 10.3389/fendo.2022.892005

**Published:** 2022-06-30

**Authors:** Bo Pang, Qiong Wang, Min Yang, Mei Xue, Yicheng Zhang, Xiangling Deng, Zhixin Zhang, Wenquan Niu

**Affiliations:** ^1^ Graduate School, Beijing University of Chinese Medicine, Beijing, China; ^2^ International Medical Services, China-Japan Friendship Hospital, Beijing, China; ^3^ Department of Pediatrics, China-Japan Friendship Hospital, Beijing, China; ^4^ Institute of Clinical Medical Sciences, China-Japan Friendship Hospital, Beijing, China

**Keywords:** precocious puberty, school children, machine learning and deep learning, prediction performance, top factor

## Abstract

**Background and Objectives:**

As the worldwide secular trends are toward earlier puberty, identification of contributing factors for precocious puberty is critical. We aimed to identify and optimize contributing factors responsible for onset of precocious puberty *via* machine learning/deep learning algorithms in girls.

**Methods:**

A cross-sectional study was performed among girls aged 6-16 years from 26 schools in Beijing based on a cluster sampling method. Information was gleaned online *via* questionnaires. Machine/deep learning algorithms were performed using Python language (v3.7.6) on PyCharm platform.

**Results:**

Of 11308 students enrolled, there are 5527 girls, and 408 of them had experienced precocious puberty. Training 13 machine learning algorithms revealed that gradient boosting machine (GBM) performed best in predicting precocious puberty. By comparison, six top factors including maternal age at menarche, paternal body mass index (BMI), waist-to-height ratio, maternal BMI, screen time, and physical activity were sufficient in prediction performance, with accuracy of 0.9530, precision of 0.9818, and area under the receiver operating characteristic curve (AUROC) of 0.7861. The performance of the top six factors was further validated by deep learning sequential model, with accuracy reaching 92.9%.

**Conclusions:**

We identified six important factors from both parents and girls that can help predict the onset of precocious puberty among Chinese girls.

## Introduction

The incidence and prevalence of precocious puberty are rising rapidly during recent decades, and differ greatly between genders around the global (1, 2). In Denmark, there was a 33-fold increase in annual incidence of premature thelarche from 1998 at 0.07 per 10,000 girls to 2007 at 2.24 per 10 000 girls, and contrastingly in boys a 15-fold increase in diagnosis of central precocious puberty was seen during this period (from 0.1 per 10 000 to 2.1 per 10 000) (3). In a separate survey from China, the adjusted prevalence of precocious puberty was estimated to be 6.19% (girls: 11.47% and boys: 3.26%) in 2019 (2). One major reason behind these alarming numbers is the worldwide secular trends toward earlier puberty. Evidence from a comprehensive meta-analysis showed that age at thelarche decreased 3 months per decade from 1977 to 2013 (4). With a great improvement in living standards and nutritional status in school-aged children, the issue of precocious puberty has received scientific attention, and we must expand this attention to develop preventive strategies to curb this global issue.

Observational studies focusing on the risk profiles of precocious puberty in school-aged children are very limited in the literature (5–8). For instance, obesity and central obesity in children were identified as promising risk factors for the development of earlier pubertal (8). In a separate study, vitamin D deficiency was associated with over 3-fold increased risk of precocious puberty in girls (6). The development of precocious puberty is a complex process, and its manifestations often involve concurrence of multiple factors, with each factor bearing a small contribution. In this context, identifying potential contributing factors and characterizing their internal relationship represents a practical strategy to inform parents and public health practitioners to delay or prevent early onset of precocious puberty. To this end, predictive models or tools are recommended to risk-stratify precocious puberty among children. However, the majority of published models or tools that are developed by traditional statistical methods are based on the “relative independence” assumption of different factors. In most cases, this assumption is untenable, as predictive factors usually act in a synergistic – instead of independent – manner, leading to biased estimation between exposures and outcomes.

To solve this methodological issue and yield more information for future studies, we identified and optimized the panel of contributing factors susceptible to onset of precocious puberty in Chinese girls aged 6-16 years by using the widely-used machine learning and deep learning algorithms. To facilitate practical application, a predictive tool using the minimal number of contributing factors and the optimal learning algorithm was proposed.

## Methods

### Study Design

This study follows the rationales of a cross-sectional design based on a stratified cluster random sampling strategy. Online survey was conducted at a suburb district (Pinggu) of Beijing during the first month of 2022. The design and implementation of this survey, in accordance with the principles of the Declaration of Helsinki, was approved the Ethics Committees of Beijing University of Chinese Medicine. The parents and supervisors of each assessable students gave informed consent prior to participation in this survey.

### Study Participants

Students from total 26 schools, which were randomly selected from Pinggu district, formed study participants of this survey. Out of 26 schools, 8 were primary schools, and 18 were middle schools. All students were initially deemed eligible for inclusion if they were not diagnosed or reported to experience severe endocrine disorders, which included but not limited to hyperthyroidism, hypothyroidism and diabetes mellitus.

### Questionnaire

An electronic questionnaire was designed, and it was *a priori* tested to be appropriate and valid based on the reliability coefficient α exceeding 0.85. For the sake of convenience, questionnaire was generated in the form of QR code that can be easily recognized by common smart phones on the market by the “Wenjuanxing” (available at the website https://www.wenjuan.com), an online crowdsourcing platform in mainland China.

### Data collection

This valid questionnaire was distributed by school teachers in charge to the parents or supervisors of each student to collect data from both students and their parents, as well as their grandparents and grandparents-in-law.

In total, parents or supervisors of 11633 students received this questionnaire, and 11308 of them responded. Given the fact that the prevalence of precocious puberty differs by gender, only 87 of 5781 boys (1.5%) were found to experience precocious puberty in this study population. To derive a reliable estimate, the present analysis was restricted to girls only. There were 5567 questionnaires filled by the parents or supervisors of girls. After excluding 40 questionnaires with missing important information, data from 5527 valid questionnaires were analyzed finally.

### Quality Control

Because data in this survey were gleaned from online questionnaires, it is essential to appraise the quality. Before circulating our questionnaires, each item was explained in details to teachers in charge by trained staff. The questionnaire was circulated by school teachers to the parents or supervisors of students under survey. In the case of any uncertainty when filling in the questionnaire, trained staff were contacted by teachers for confirmation. Labeled data were downloaded from the “Wenjuanxing” platform into the Microsoft Office Excel™ file. All data were double checked by trained staff to ensure accuracy. In the case of missing data or data with extreme values, teachers in charge were contacted by re-inviting the parents or Supervisors to provide or validate these data.

### Survey Data From Students

Data on birth year and month, sex, nationality, birth weight, birth body length, gestational week, time spent on physical activity and screen, daily sitting time, time to night sleep, sleep duration and eating speed were collected from students under study. In addition, dietary preferences including frequency of eating fiber-rich, out-of-season, meat, plant protein, tonic or sweet foods, snacks, milk products, foods with preservatives or fast foods were recorded. Lifestyle habits that may affect puberty onset were also recorded, including consuming night meal, sleeping with the lights on, monophagia, frequent use of plastic tableware, and exposures to adult cosmetics. Body height (to the nearest 0.1 cm), body weight (to the nearest 0.1 kg), and waist circumference (to the nearest 0.1 cm) were measured by trained physicians. Waist circumference was measured at 2 cm above the umbilicus.

### Survey Data From Parents and Grandparents

Data collected from parents and grandparents included birth year, sex, body height and weight, education, family income, maternal age at menarche, baring age of both parents, pregnancy order, delivery order, history of using assisted reproductive technology, delivery mode, breastfeeding duration, infancy feeding ways, and age of adding complementary food. The number of two parents and four grandparent who were diagnosed to have hypertension or diabetes mellitus was summed meanwhile.

### Precocious Puberty Definition

Precocious puberty was defined according to the guidelines recommended by the Chinese Medical Association Pediatrics Branch in 2015 (9) and the Ministry of Health of the People’s Republic of China in 2011 (10). Girls with breast development before 8 years of age and menarche before 10 years of age are considered to experience precocious puberty.

As this survey was undertaken in schools and due to the strict prevention and control for COVID-19 in China, it is difficult to examine the physical status of students on spot. Self-observation and visual assessment methods suitable for survey were adopted to determine whether girls had precocious puberty (11). In detail, the standards for breast development are that the breasts are hill-shaped and palpated with nodules inside. To reduce or avoid information bias, the methods were explained in detail by trained staff to teachers in charge, as well as the parents and grandparents of each girl in the survey.

### Definitions of Survey Data

Body mass index (BMI) was calculated as follows: weight (kg)/height (m^2^). Waist-to-height ratio (WHtR) was calculated as waist (cm) divided by height (cm). Central obesity was defined as WHtR ≥0.47 in girls (12). Duration of sleeping, physical activity, sitting, and screening was recorded in hours and separately calculated as the sum of time on working days × 5 and time on weekends × 2 divided by 7. Eating speed was recorded in minutes and calculated as the average time of breakfast, lunch and supper within a day. All types of dietary preferences and habits that may affect puberty onset were categorized into 4 groups: every day, 3-5 times weekly, 1-2 times weekly and none or occasionally. Fiber-rich foods referred to grains, seasonal vegetables and fruits. Night meal was defined as eating foods within 2 hours before bedtime. Screen time was defined as sum of time spent on watching television, using computers and mobile phones, and the other kinds of electronic devices with screens.

Maternal BMI and paternal BMI were calculated from self-reported weight and height. Education was classified into Bachelor’s (or equivalent) degree or above, high (or equivalent) school degree, and junior high school degree or below. Household income was categorized as <100,000, 100,000-300,000 and >300,000 RMB per year. Delivery mode included natural labor and cesarean section. Breastfeeding duration and age of adding complementary foods were recorded in months. Infancy feeding ways included exclusive breastfeeding, mixed feeding, and artificial feeding.

### Statistical Analyses

Data were analyzed using the PyCharm (Edition 2018.1 x64) embedded by the Python (Python Software Foundation) software (Version 3.7.6) under the Windows 10 system. Variables were retained for analysis if the percentage of missing rows is less than 30%. Missing data were filled in by multiple imputation method using the “mice” package in the R environment (Version 4.1.1).

As this survey was performed at 26 schools, the degree of difference between schools was appraised by the intraclass correlation coefficient (ICC) (13). The ICC statistic ranges from zero to unity, and if this statistic equals to zero, the variance in questionnaire is not due to variation between schools.

All study girls were grouped according to the absence or presence of precocious puberty features. Descriptive statistics were expressed as mean (standard deviation) or median (interquartile range) for continuous variables, and as number (percent) for categorical variables. Deviation from normal distribution was tested from skewness and kurtosis statistics. Differences in these variables between girls with and without precocious puberty were quantified by using the t test, rank-sum test or χ^2^, when appropriate. Two-sided probability was deemed statistically significant if it is less than 0.05.

All valid variables were fed to machine learning algorithms. To minimize overfitting, data from 5527 girls were randomized into the training set and the validation set at a ratio of 6:4. Due to the low ratio of precocious puberty, the purpose of this classification choice is to ensure that there are sufficient number of cases with precocious puberty in both training and validation sets.

In this study, besides the traditional logistic regression algorithm, additional 12 different machine learning algorithms were employed, including decision tree, Adaboost decision tree, support vector machine (SVM), random forest, K-nearest neighbor (KNN), gradient boosting machine (GBM), extreme gradient boosting (Xgbc), light gradient boosting machine (LGBM), Gaussian naive Bayes (gNB), multinomial naive Bayes (mNB), Bernoulli naive Bayes (bMB) and multi-layer perceptron (MLP). The machine learning algorithms performed supervised learning in given data, adjusting for the magnitude and direction of model error to improve prediction accuracy. Data from the training set were first fed to each machine learning algorithm, and data from the validation set were used to test prediction performance of these algorithms. Prediction performance was assessed from five diverse aspects, namely, accuracy, precision, recall, F1 score, and the area under the receiver operating characteristic curve (AUROC). Based on the five aspects, the optimal machine learning algorithm was selected. By definition, accuracy refers to the rate of correct prediction, and precision measures the ability to target actual positive observations. Recall reflects the capability to predict actual positivity correctly. F1 score, calculated as the harmonic mean between precision and recall, takes both false positives and false negatives into account. AUROC is proposed as a summarized accuracy index, with a higher value indicating a higher probability of having the characteristic under study.

With the use of optimal machine learning algorithm, the minimum number of top variables were determined by prediction capability. The prediction capability of cumulative variables was reflected by AUROC, accuracy, and precision, upon which, the top variables was ranked in an ascending order. For clinical application, the minimal number of top variables that can achieve maximum predictive performance was ascertained. Performance of this minimal number of top variables in predicting onset of precocious puberty in girls was further appraised by accuracy and loss using the deep learning sequential model. For comparison, deep learning sequential model was performed using 3 different optimizers [adaptive moment estimation (Adam), root mean square prop (RMSprop), and stochastic gradient descent (SGD)].

To reflect the degree of prediction directly, the top variables identified above were incorporated into the Logistic regression model. The effect-size estimates are expressed as odds ratio (OR) with 95% confidence interval (CI).

## Results

### Between-School Variation Appraisal

The ICC statistic for all items in our questionnaire was extremely low (less than 0.044), meaning that there was low likelihood of clustering within schools, as well as low likelihood of difference in items between schools.

### Baseline Characteristics

**Table 1** shows the distributions and comparisons of 5527 girls (mean age: 10.88 years, range: 6-16 years) stratified by the presence and absence of precocious puberty. 408 girls were defined to experience precocious puberty, with the prevalence rate of 7.4% in this girl population.

**Table 1:** The baseline characteristics of school girls stratified by the presence of precocious puberty.

Survey factors		Absence of precocious puberty	Presence of precocious puberty	P
		(n = 5119)	(n = 408)	
Age (months)		130.0 [106.0, 157.0]	131.0 [111.0, 152.00]	0.573
Ethnicity (%)	Han	4888 (95.5)	393 (96.3)	0.892
	Man	143 (2.8)	9 (2.2)	
	Hui	12 (0.2)	0 (0.0)	
	Others	76 (1.5)	6 (1.5)	
WHtR		0.43 [0.39, 0.48]	0.45 [0.40, 0.50]	<0.001
BMI		18.07 [15.61, 21.23]	20.05 [17.79, 23.29]	<0.001
Fiber-rich foods (%)	None or occasionally	90 (1.8%)	11 (2.7%)	0.095
	1-2 times weekly	793 (15.5%)	77 (18.9%)	
	3-5 times weekly	1446 (28.2%)	117 (28.7%)	
	Every day	2790 (54.5%)	203 (49.8%)	
Out-of-season foods (%)	None or occasionally	688 (13.4%)	53 (13.0%)	0.041
	1-2 times weekly	1845 (36.0%)	174 (42.6%)	
	3-5 times weekly	1399 (27.3%)	105 (25.7%)	
	Every day	1187 (23.2%)	76 (18.6%)	
Animal protein foods (%)	None or occasionally	60 (1.2%)	7 (1.7%)	0.565
	1-2 times weekly	834 (16.3%)	62 (15.2%)	
	3-5 times weekly	1643 (32.1%)	139 (34.1%)	
	Every day	2582 (50.4%)	200 (49.0%)	
Plant protein foods (%)	None or occasionally	424 (8.3%)	38 (9.3%)	0.357
	1-2 times weekly	2038 (39.8%)	175 (42.9%)	
	3-5 times weekly	1436 (28.1%)	111 (27.2%)	
	Every day	1221 (23.9%)	84 (20.6%)	
Milk products (%)	None or occasionally	179 (3.5%)	6 (1.5%)	0.112
	1-2 times weekly	755 (14.7%)	55 (13.5%)	
	3-5 times weekly	1314 (25.7%)	108 (26.5%)	
	Every day	2871 (56.1%)	239 (58.6%)	
Tonic foods (%)	None or occasionally	4218 (82.4%)	352 (86.3%)	0.077
	1-2 times weekly	517 (10.1%)	37 (9.1%)	
	3-5 times weekly	181 (3.5%)	12 (2.9%)	
	Every day	203 (4.0%)	7 (1.7%)	
Food with preservatives (%)	None or occasionally	2831 (55.3%)	219 (53.7%)	0.094
	1-2 times weekly	1730 (33.8%)	153 (37.5%)	
	3-5 times weekly	366 (7.1%)	29 (7.1%)	
	Every day	192 (3.8%)	7 (1.7%)	
Fast foods (%)	None or occasionally	2357 (46.0%)	187 (45.8%)	0.335
	1-2 times weekly	2416 (47.2%)	195 (47.8%)	
	3-5 times weekly	233 (4.6%)	22 (5.4%)	
	Every day	113 (2.2%)	4 (1.0%)	
Snacks (%)	None or occasionally	909 (17.8%)	58 (14.2%)	0.087
	1-2 times weekly	2907 (56.8%)	244 (59.8%)	
	3-5 times weekly	914 (17.9%)	83 (20.3%)	
	Every day	389 (7.6%)	23 (5.6%)	
Sweet foods (%)	None or occasionally	885 (17.3%)	57 (14.0%)	0.126
	1-2 times weekly	3001 (58.6%)	244 (59.8%)	
	3-5 times weekly	932 (18.2%)	88 (21.6%)	
	Every day	301 (5.9%)	19 (4.7%)	
Eating speed (minutes)		16.67 [13.33, 20.00]	16.67 [13.33, 20.00]	0.063
Night meal (%)	None or occasionally	2653 (51.8)	219 (53.7)	0.02
	1-2 times weekly	1482 (29.0)	135 (33.1)	
	3-5 times weekly	533 (10.4)	29 (7.1)	
	Every day	451 (8.8)	25 (6.1)	
Sleep with lights on (%)	None or occasionally	4426 (86.5)	355 (87.0)	0.99
	1-2 times weekly	298 (5.8)	24 (5.9)	
	3-5 times weekly	126 (2.5)	9 (2.2)	
	Every day	269 (5.3)	20 (4.9)	
Monophagia (%)	None or occasionally	2552 (49.9%)	239 (58.6%)	0.005
	1-2 times weekly	1534 (30.0%)	110 (27.0%)	
	3-5 times weekly	561 (11.0%)	32 (7.8%)	
	Every day	472 (9.2%)	27 (6.6%)	
Use of plastic tableware (%)	None or occasionally	3328 (65.0%)	264 (64.7%)	0.657
	1-2 times weekly	1036 (20.2%)	88 (21.6%)	
	3-5 times weekly	311 (6.1%)	27 (6.6%)	
	Every day	444 (8.7%)	29 (7.1%)	
Cosmetics exposure (%)	None or occasionally	4650 (90.8%)	368 (90.2%)	0.038
	1-2 times weekly	301 (5.9%)	18 (4.4%)	
	3-5 times weekly	65 (1.3%)	12 (2.9%)	
	Every day	103 (2.0%)	10 (2.5%)	
Physical activity (hours per day)		1.29 [1.00, 1.57]	1.00 [0.86, 1.57]	0.002
Sitting duration (hours per day)		5.86 [3.43, 7.43]	6.29 [4.14, 7.43]	0.032
Screen time (hours per day)		1.29 [0.64, 1.57]	1.29 [0.89, 2.00]	<0.001
Sleep duration (hours per day)		9.00 [8.29, 9.29]	8.71 [8.29, 9.29]	0.067
Fall asleep time (hours per day)		10.00 [9.00, 10.00]	10.00 [9.50, 10.00]	0.008
Pregnancy order (%)	1	3404 (66.8%)	269 (66.1%)	0.437
	2	1260 (24.7%)	96 (23.6%)	
	3	340 (6.7%)	30 (7.4%)	
	4	72 (1.4%)	10 (2.5%)	
	5	21 (0.4%)	2 (0.5%)	
Delivery order (%)	1	4303 (84.4%)	363 (89.0%)	0.096
	2	710 (13.9%)	41 (10.0%)	
	3	67 (1.3%)	3 (0.7%)	
	4	17 (0.3%)	1 (0.2%)	
Delivery mode (%)	Vaginal delivery	2558 (50.0%)	190 (46.6%)	0.198
	Cesarean section	2561 (50.0%)	218 (53.4%)	
Assisted reproductive technology (%)	Unused	5043 (98.5%)	401 (98.3%)	0.671
	Used	76 (1.5%)	7 (1.7%)	
Gestational week		39.00 [38.00, 40.00]	39.00 [38.00, 40.00]	0.006
Birth weight (kg)		3.30 [3.00, 3.60]	3.30 [3.00, 3.50]	0.038
Birth body length (cm)		50.00 [50.00, 52.00]	50.00 [50.00, 52.00]	0.922
Bearing age of father		27.58 [25.67, 30.08]	27.33 [25.75, 29.50]	0.529
Bearing age of mother		26.58 [24.50, 28.92]	26.33 [24.33, 28.33]	0.218
Infancy feeding (%)	Breastfeeding	3027 (59.1%)	226 (55.4%)	0.217
	Mixed feeding	1523 (29.8%)	127 (31.1%)	
	Artificial feeding	569 (11.1%)	55 (13.5%)	
Breastfeeding duration (months)		8.00 [0.00, 13.00]	7.00 [0.00, 12.00]	0.123
Time to add complementary (months)		6.00 [6.00, 7.00]	6.00 [6.00, 6.00]	0.008
Maternal age at menarche		13.00 [12.00, 14.00]	13.00 [12.00, 14.00]	<0.001
Paternal BMI		25.43 [23.39, 27.76]	25.95 [23.75, 28.49]	0.005
Maternal BMI		22.92 [20.82, 25.39]	23.90 [21.48, 26.44]	<0.001
Number of relatives with hypertension (%)	0	2420 (47.3%)	149 (36.5%)	<0.001
	1	1186 (23.2%)	120 (29.4%)	
	2	939 (18.3%)	88 (21.6%)	
	3	412 (8.0%)	34 (8.3%)	
	4	162 (3.2%)	17 (4.2%)	
Number of relatives with diabetes (%)	0	3471 (67.8%)	266 (65.2%)	0.131
	1	1216 (23.8%)	96 (23.5%)	
	2	348 (6.8%)	40 (9.8%)	
	3	63 (1.2%)	3 (0.7%)	
	4	21 (0.4%)	3 (0.7%)	
Paternal education (%)	Junior high school degree or below	803 (15.7%)	54 (13.2%)	0.167
	High school degree	1857 (36.3%)	139 (34.1%)	
	Bachelor’s degree or above	2459 (48.0%)	215 (52.7%)	
Maternal education (%)	Middle school degree or below	806 (15.7%)	48 (11.8%)	0.074
	High school degree	1490 (29.1%)	118 (28.9%)	
	Bachelor’s degree or above	2823 (55.1%)	242 (59.3%)	
Household income (RMB per year)	<100,000	2420 (47.3%)	162 (39.7%)	0.003
	100,000-300,000	2299 (44.9%)	200 (49.0%)	
	>300,000	400 (7.8%)	46 (11.3%)	

Continuous data are expressed as mean (standard deviation) in normal distributions and median [interquartile range] in skewed distributions. Categorical data are expressed as count (percentage). For continuous data, P for comparison between girls with non-precocious puberty or precocious puberty was derived by t test for normally distributed data, by rank-sum test for skewed data, and by χ2 test for categorical data. WHtR, waist-to-height ratio; BMI, body mass index.

### Selecting the Optimal Machine Learning Algorithm

**Figure 1:**
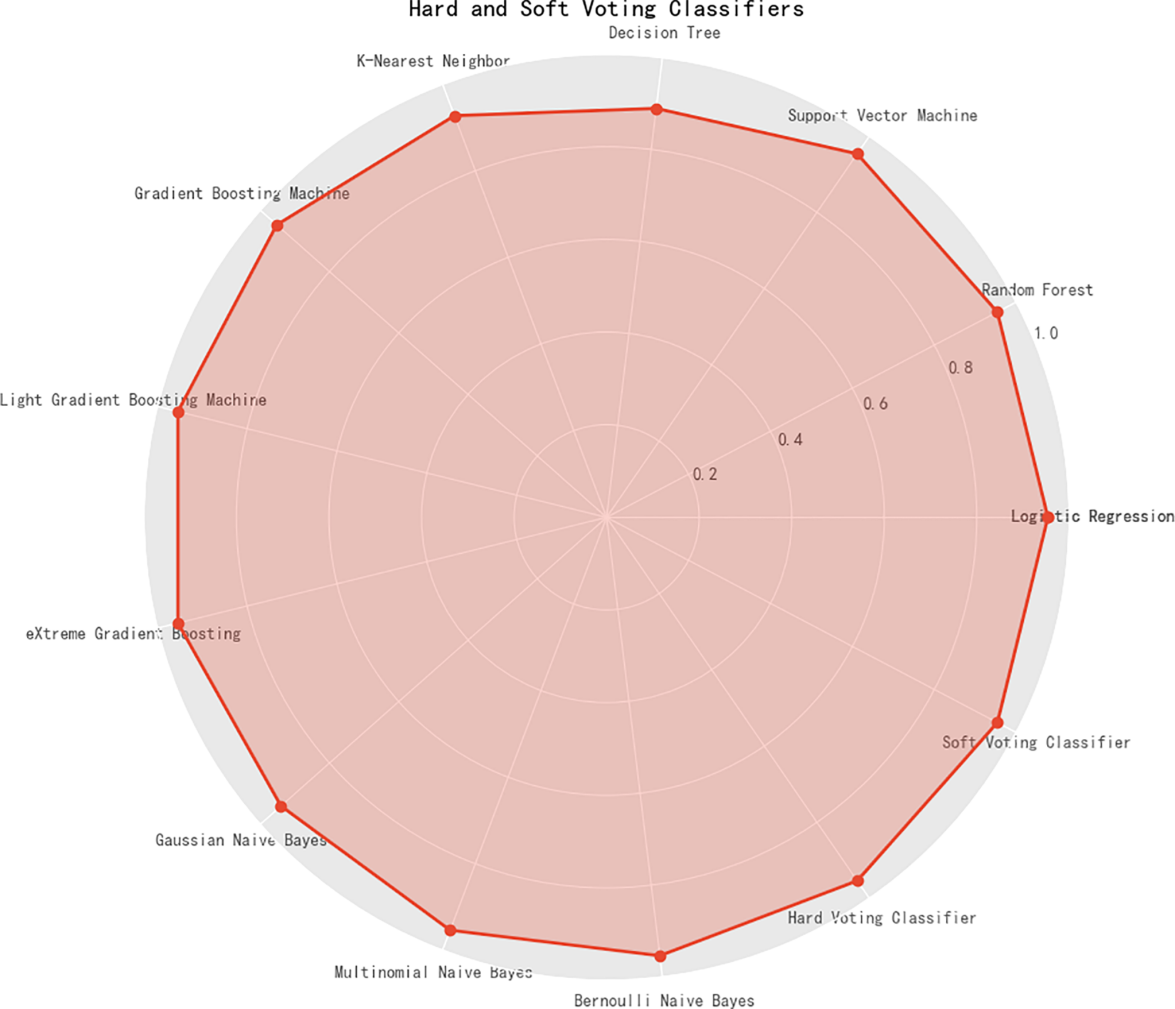
The prediction accuracy of 13 machine learning algorithms, along with hard and soft voting classifiers.

**Figure 2:**
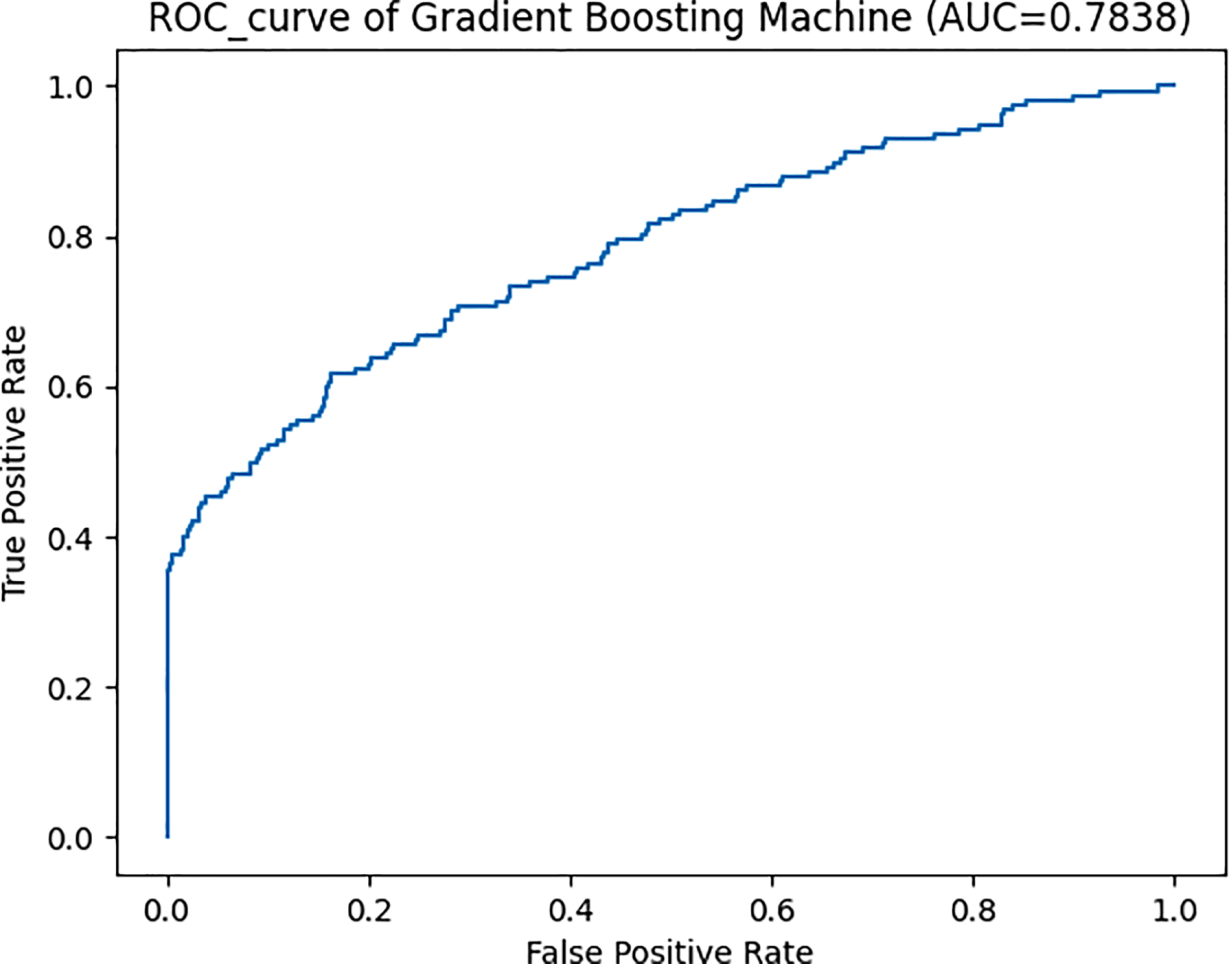
The area under the receiver operating characteristic curve (AUROC) of gradient boosting machine algorithm for the prediction of precocious puberty. AUC, area under the receiver operating characteristic curve; ROC, receiver operating characteristic curve.

**Figure 1** shows the prediction accuracy of 13 different machine learning algorithms evaluated in this study. **Table 2** compares the performance of these algorithms from five aspects. After comparison, the algorithm GBM performed the best, with the AUROC of 0.784 (**Figure 2**), the accuracy of 0.952, the precision of 0.932, the recall of 0.350, and the F1 score of 0.509. The prediction model for precocious puberty was therefore constructed using the GBM algorithm.

**Table 2:** The prediction performance of 13 machine learning algorithms from accuracy, precision, recall, F1 score and AUROC aspects for precocious puberty.

Algorithms	Accuracy	Precision	Recall	F1 score	AUROC
Logistic regression	0.9534	1.0000	0.3439	0.4118	0.7799
Decision tree	0.8892	0.2864	0.3758	0.3251	0.6521
Adaboost decision tree	0.9507	0.9286	0.3312	0.4883	0.7724
Support vector machine	0.9534	1.0000	0.3439	0.5118	0.7576
Random forest	0.9534	1.0000	0.3439	0.5118	0.7513
K-nearest neighbor	0.9290	0.0000	0.0000	0.0000	0.5743
Gradient boosting machine	0.9521	0.9322	0.3503	0.5093	0.7838
Extreme gradient boosting	0.9539	1.0000	0.3503	0.5189	0.6752
Light gradient boosting machine	0.9539	1.0000	0.3503	0.5189	0.7822
Multi-layer perceptron	0.9290	0.0000	0.0000	0.0000	0.5057
Gaussian naive Bayes	0.9398	0.6395	0.3503	0.4527	0.7658
Multinomial naive Bayes	0.9534	1.0000	0.3439	0.5118	0.7202
Bernoulli naive Bayes	0.9534	1.0000	0.3439	0.5118	0.6960

AUROC, area under the receiver operating characteristic curve.

### Selecting the Minimal Number of Contributors

**Figure 3:**
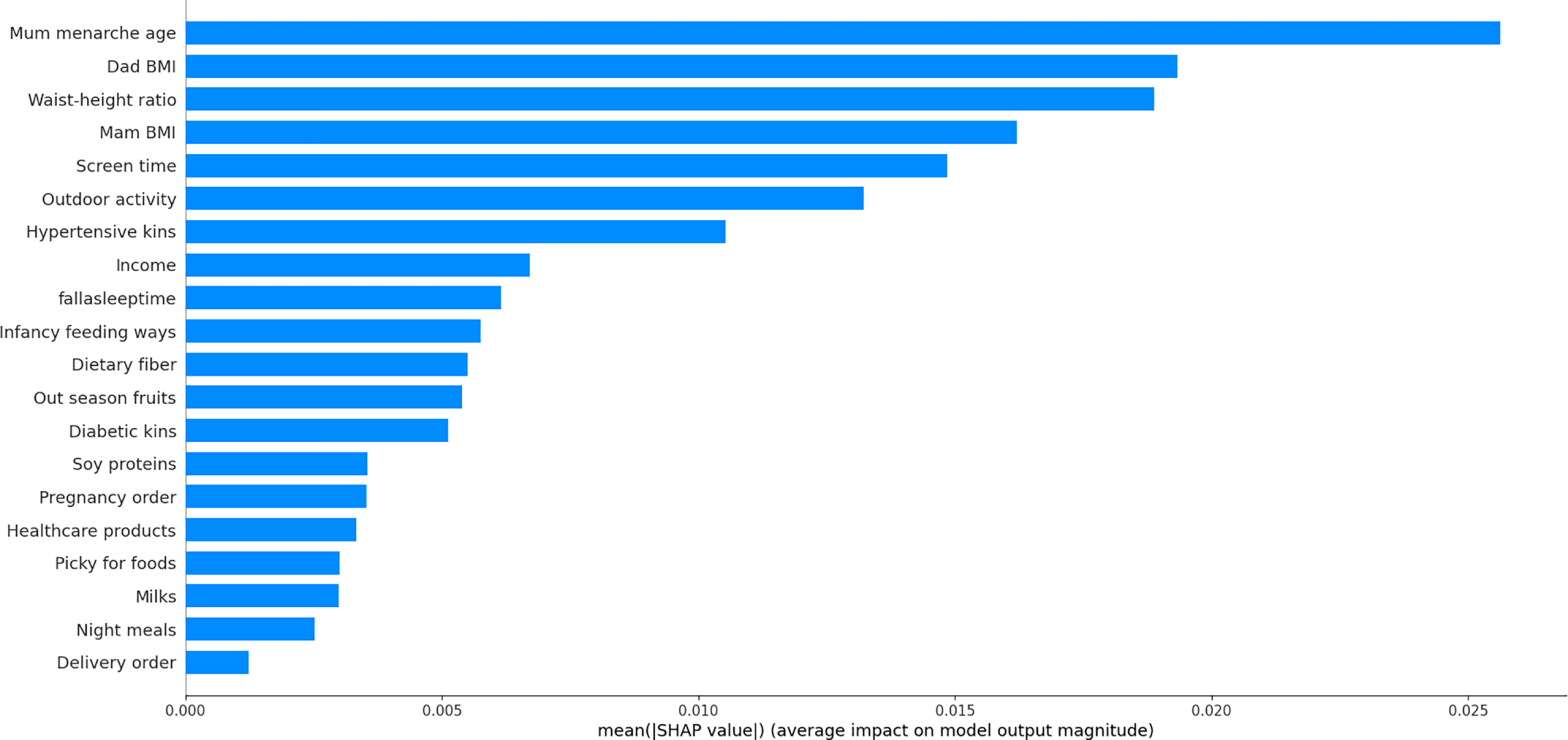
Top 20 factors for predicting precocious puberty in a descending order of importance. BMI, body mass index.

The importance of each variable under study in predicting precocious puberty was estimated and ranked in an ascending order, and that of the top 20 variables is illustrated in **Figure 3**. The cumulative contribution of top 10 variables is assessed in **Table 3**, and by comparison the prediction capability of top six variables was comparable with that of all 45 variables under study (AUROC: 0.7861; accuracy: 0.9534; precision: 1.0000). The six variables included maternal age at menarche, paternal BMI, WHtR, maternal BMI, screen time, and physical activity.

**Table 3:** The areas under the receiver operating curve (AUROC), accuracy and precision with the cumulating number of top ten factors in an ascending order.

Number of top ten factors in rank	AUROC	Accuracy	Precision
1	0.6720	0.9534	1.0000
2	0.6862	0.9534	1.0000
3	0.7137	0.9534	1.0000
4	0.7202	0.9534	1.0000
5	0.7457	0.9525	0.9642
6	0.7861	0.9530	0.9818
7	0.7863	0.9530	0.9818
8	0.7852	0.9525	0.9333
9	0.7806	0.9520	0.9473
10	0.7722	0.9516	0.9310

### Performance Assessment Using Deep Learning Model

As presented in **Table 4**, the prediction performance was compared between top 6 variables and all study variables under the deep learning sequential model. By using three optimizers, the performance as assessed by model accuracy and model loss was comparable between the two sets of variables (for accuracy: 94.12% using all variables and 92.90% using top six variables; for loss: 26.05% using all variables and 25.66% using top six variables in the validation set), which further reinforced the prediction capability of the top six variables selected by machine learning algorithms.

**Table 4:** Model loss and accuracy of deep learning sequential model in both training and testing groups.

Optimizers	Training group		Testing group	
	Loss	Accuracy	Loss	Accuracy
All factors				
Adam	11.68%	96.32%	31.97%	93.76%
RMSprop	15.30%	95.52%	26.05%	94.12%
SGD	15.86%	95.75%	23.69%	93.80%
Top 6 factors				
Adam	26.31%	92.57%	25.71%	92.90%
RMSprop	24.06%	93.51%	25.66%	92.90%
SGD	25.20%	93.07%	25.63%	92.90%

Adam, adaptive moment estimation; RMSprop, root mean square prop; SGD, stochastic gradient descent.

### Risk Estimates of the Top 6 Variables Using Logistic Regression Model

The top 6 variables identified above was incorporated into the Logistic regression model to estimate the effect-size of prediction (**Table 5**). Consistent with the above findings, the top 6 variables were significantly associated with the onset of precocious puberty.

**Table 5:** The risk prediction of top 6 variables for precocious puberty using the Logistic regression model.

Top 6 factors	OR (95% CI)	P
Maternal age at menarche	0.74 (0.69, 0,80)	<0.001
Paternal BMI	1.04 (1.02, 1.07)	0.003
Waist-to-height ratio	1.31 (1.15, 1.49)	<0.001
Maternal BMI	1.06 (1.03, 1.10)	<0.001
Screen time	1.12 (1.04, 1.22)	0.003
Physical activity	0.78 (0.66, 0.93)	0.006

OR, odds ratio; 95% CI, 95% confidence interval.

## Discussion

This is a cross-sectional, large survey in school girls aged 6-16 years from Beijing to identify and optimize contributing factors susceptible to precocious puberty. The key findings of this study are the contribution of six top factors from both parents and girls under the optimal GBM algorithm to the prediction of precocious puberty, which can not only help inform parents to prevent or postpone the earlier puberty of their daughters, but also suggest healthcare professionals to launch education reforms, such as weight control and outdoor activity recommendation. Importantly, the incorporation of the six top factors in GBM algorithm can be developed as a tool to facilitate the better identification of school girls who are at high risk for the future experience of precocious puberty and might benefit from early lifestyle or medical interventions. To the best of our knowledge, this is thus far the first attempt of using machine learning and deep learning techniques to characterize the risk profiles of precocious puberty in school girls.

Recently, the problem of precocious puberty constitutes a substantial health burden around the global (14). Several studies have shown that the incidence of precocious puberty have increased significantly and there are worldwide secular trends toward earlier puberty (2, 3, 11). Some studies have shown that early puberty is associated with cognitive function decline in children with Cushing disease (15). Another study using Mendelian randomization method provided evidence for the causal detrimental impact of early puberty on asthma (16). In a separate population study, early onset of breast development was found to be associated with high risk of the presence of depression (17). In addition, animal studies indicated puberty as an important developmental period for the establishment of adipose tissue mass and metabolic homeostasis (18). Given the high burden of precocious puberty, it is imperative to gain an understanding of its risk profiles for informing future gender-specific public health recommendations.

There is evidence that the development of precocious puberty is a multistep, multifactorial progress, to which inherited, environmental, and lifestyle-related factors contribute together (11). In most cases, the contribution of each factor is dependent on the presence of other factors, and this dependence usually follows a nonlinear manner. Despite there are good reasons to adopt traditional statistical approaches such as logistic regression, they do have limited power for modeling high-order non-linear patterns of interaction. Hence, success in characterize the risk profiles of precocious puberty will hinge on our ability to address nonlinearities and interactions between exposures and outcomes. To shed some light, besides the traditional logistic regression, we also employed 12 additional machine learning algorithms to compare their prediction performance from various aspects. These machine learning algorithms have shown great promise in application to many areas in clinical practice and health care, such as assisted disease diagnosis, clinical outcome prediction and automated image interpretation (19–22). By comparison, we found the prediction performance of GBM algorithm is best, and this algorithm focuses on improved prediction by combining information from many variables that individually may not be significant but together are very informative; of less concern is the functional form of any one variable (23). The GBM algorithm builds regression trees on all factors in parallel to take adequate utilization of all variables. On the other hand, the GBM algorithm might have the risk of overfitting. The possibility of overfitting can be checked through the accuracy of prediction in the validation set. What’s more, the six top factors, including age at menarche, paternal BMI, WHtR, maternal BMI, screen time, and physical activity were teased out from 45 potential factors in this study and their prediction performance was not inferior to all factors involved. The most important factor identified in this study, age at menarche, was supported by other studies (24, 25).

Besides, WHtR was found to be associated with the significant risk of precocious puberty in this study, consistent with the findings of previous studies (8). Relative to BMI, WHtR is an indicator of central obesity and is less correlated with age (12, 26). There is evidence that excessive visceral fat accumulation, manifested as central obesity, can impair insulin sensitivity and even result in insulin resistance (27), and reduced insulin sensitivity interferes with leptin signaling, which plays in important role in puberty initiation (28). Our findings highlight the suitability of monitoring WHtR among school girls in predicting the risk of precocious puberty. Another aspect that merits discussion is the contributory role of parental obesity in predisposition to precocious puberty in offspring, which was consolidated by other studies (29).

Moreover, it is increasingly recognized that physical activity and screen time are two factors precipitating the onset of puberty. As stated by a systematic review, menarche of athletes with high-intensity physical activity delayed 1.13 years compared to non-athlete girls (30). In a separate study, aerobic exercise was reported to delay the progression of puberty in students with central precocious puberty by increasing adiponectin levels (31). On the other hand, screen time may alter eating habits (32), and advertisements through screens would influence dietary choices (33). Notably, the incidence of precocious puberty increased significantly during the COVID-19 lockdown due to the prolonged usage of electronic devices (34). There is evidence that prolonged screen time can reduce insulin sensitivity and lead to high fasting insulin levels in girls (35). We agree that further validations of our findings in other independent groups are necessary to confirm the implication of six factors identified in the development of precocious puberty in school girls.

It is also worth noting that the contribution of these six factors to precocious puberty is not independent, and they might act in a synergistic manner. Although the GBM was selected as the best algorithm in predicting precocious puberty, the modeling process is less transparent and is harder to interpret, restricting clinical understanding and implementation in practice. As such, a high standard of model transparency is required to allow parents and students, as well as healthcare professionals to make informed decisions.

### Limitations

Several limitations should be acknowledged for the present study. Firstly, the cross-sectional nature of this survey limited further interpretation of possible causality between important factors identified and precocious puberty. Secondly, despite much efforts are made to ensure data quality, information bias cannot be fully ruled out in our questionnaires. Thirdly, puberty is a complex biological process of sexual progression affected by a dynamic panel of risk factors, and incorporation of more lifestyle-relevant factors of students is of added interest. Fourthly, all students enrolled are school-aged girls living in Beijing, and extrapolation of our findings to other populations should be made with caution. Fifthly, by means of questionnaire, it is only possible to appraise the presence of precocious puberty, rather than to distinguish the types of precocious puberty, such as central precocious puberty. We agree that further studies are warranted to identify and optimize the contributing factors susceptible to the onset of central precocious puberty.

### Conclusions

*Via* a comprehensive analysis, we have identified six important factors from both parents and students under the optimal GBM algorithm that can help predict the risk of precocious puberty in Chinese girls. Further explorations on other potential factors, especially for school boys and possible molecular mechanisms of precocious puberty are encouraging and essential.

## Data Availability Statement

The raw data supporting the conclusions of this article will be made available by the authors, without undue reservation.

## Ethics Statement

The studies involving human participants were reviewed and approved by Ethics Committees of Beijing University of Chinese Medicine. Written informed consent to participate in this study was provided by the participants’ legal guardian/next of kin.

## Author Contributions

ZZ planned and designed the study, as well as directed its implementation. ZZ and WN drafted the protocol. QW, BP, MY, MX, YZ, and XD obtained statutory and ethics approvals. QW and BP contributed to data acquisition. BP and WN conducted statistical analyses. QW, BP, MY and MX did the data preparation and quality control. BP and WN wrote the manuscript. All authors read and approved the final manuscript prior to submission. All authors contributed to the article and approved the submitted version.

## Conflict of Interest

The authors declare that the research was conducted in the absence of any commercial or financial relationships that could be construed as a potential conflict of interest.

## Publisher’s Note

All claims expressed in this article are solely those of the authors and do not necessarily represent those of their affiliated organizations, or those of the publisher, the editors and the reviewers. Any product that may be evaluated in this article, or claim that may be made by its manufacturer, is not guaranteed or endorsed by the publisher.

## References

[1] Phillip M Lazar L. Precocious Puberty: Growth and Genetics. Horm Res (2005) 64(Suppl 2):56–61. doi: 10.1159/000087760 16286772

[2] Liu Y Yu T Li X Pan D Lai X Chen Y. Prevalence of Precocious Puberty Among Chinese Children: A School Population-Based Study. Endocrine (2021) 72(2):573–81. doi: 10.1007/s12020-021-02630-3 33528762

[3] Brauner EV Busch AS Eckert-Lind C Koch T Hickey M Juul A. Trends in the Incidence of Central Precocious Puberty and Normal Variant Puberty Among Children in Denmark, 1998 to 2017. JAMA Netw Open (2020) 3(10):e2015665. doi: 10.1001/jamanetworkopen.2020.15665 33044548 PMC7550972

[4] Eckert-Lind C Busch AS Petersen JH Biro FM Butler G Brauner EV. Worldwide Secular Trends in Age at Pubertal Onset Assessed by Breast Development Among Girls: A Systematic Review and Meta-Analysis. JAMA Pediatr (2020) 174(4):e195881. doi: 10.1001/jamapediatrics.2019.5881 32040143 PMC7042934

[5] Kim Y Lee NK Kim JH Ku JK Lee BK Jung HI. Association of Maxillary Dental Developmental Abnormality With Precocious Puberty: A Case-Control Study. Maxillofac Plast Reconstr Surg (2020) 42(1):30. doi: 10.1186/s40902-020-00274-3 32884928 PMC7447735

[6] Lee HS Kim YJ Shim YS Jeong HR Kwon E Hwang JS. Associations Between Serum Vitamin D Levels and Precocious Puberty in Girls. Ann Pediatr Endocrinol Metab (2014) 19(2):91–5. doi: 10.6065/apem.2014.19.2.91 PMC4114046 25077092

[7] Wen Y Liu SD Lei X Ling YS Luo Y Liu Q. Association of Paes With Precocious Puberty in Children: A Systematic Review and Meta-Analysis. Int J Environ Res Public Health (2015) 12(12):15254–68. doi: 10.3390/ijerph121214974 PMC4690910 26633449

[8] Chen C Zhang Y Sun W Chen Y Jiang Y Song Y. Investigating the Relationship Between Precocious Puberty and Obesity: A Cross-Sectional Study in Shanghai, China. BMJ Open (2017) 7(4):e014004. doi: 10.1136/bmjopen-2016-014004 PMC5566589 28400459

[9] Subspecialty Group of Endocrinologic, Hereditary and Metabolic Diseases, the Society of Pediatrics Chinese Medical Association Editorial Board, Chinese Journal of Pediatrics. Consensus on Diagnosis and Treatment of Central Precocious Puberty (2015). Chin J Pediatr (2015) 53(6):7. doi: 10.3760/cma.j.issn.0578-1310.2015.06.004 26310550

[10] China MoHotPsRo. Guidelines for the Diagnosis and Treatment of Precocious Puberty (Trial). Chin J Child Health Care (2011) 19(4):390–92. doi: CNKI:SUN:ERTO.0.2011-04-038

[11] Abreu AP Kaiser UB. Pubertal Development and Regulation. Lancet Diabetes Endocrinol (2016) 4(3):254–64. doi: 10.1016/S2213-8587(15)00418-0 PMC5192018 26852256

[12] Weili Y He B Yao H Dai J Cui J Ge D. Waist-To-Height Ratio Is an Accurate and Easier Index for Evaluating Obesity in Children and Adolescents. Obes (Silver Spring) (2007) 15(3):748–52. doi: 10.1038/oby.2007.601 17372326

[13] Donner A. A Review of Inference Procedures for the Intraclass Correlation Coefficient in the One-Way Random Effects Model. Int Stat Rev (1986) 54:67–82. doi: 10.2307/1403259

[14] Chen SY Fuldeore M Boulanger L Fraser KA Chwalisz K Marx SE. Medical Resource Use and Costs Related to Central Precocious Puberty: A Retrospective Cohort Study. Endocr Pract (2012) 18(4):519–28. doi: 10.4158/EP11293.OR 22440983

[15] Keil MF Kang JY Liu A Wiggs EA Merke D Stratakis CA. Younger Age and Early Puberty Are Associated With Cognitive Function Decline in Children With Cushing Disease. Clin Endocrinol (Oxf) (2022) 96(4):569–77. doi: 10.1111/cen.14611 PMC8897227 34668209

[16] Minelli C van der Plaat DA Leynaert B Granell R Amaral AFS Pereira M. Age at Puberty and Risk of Asthma: A Mendelian Randomisation Study. PloS Med (2018) 15(8):e1002634. doi: 10.1371/journal.pmed.1002634 30086135 PMC6080744

[17] Wang H Lin SL Leung GM Schooling CM. Age at Onset of Puberty and Adolescent Depression: "Children of 1997" Birth Cohort. Pediatrics (2016) 137(6):e20153231. doi: 10.1542/peds.2015-3231 27230766

[18] Holtrup B Church CD Berry R Colman L Jeffery E Bober J. Puberty Is an Important Developmental Period for the Establishment of Adipose Tissue Mass and Metabolic Homeostasis. Adipocyte (2017) 6(3):224–33. doi: 10.1080/21623945.2017.1349042 PMC5638358 28792785

[19] Flores AM Demsas F Leeper NJ Ross EG. Leveraging Machine Learning and Artificial Intelligence to Improve Peripheral Artery Disease Detection, Treatment, and Outcomes. Circ Res (2021) 128(12):1833–50. doi: 10.1161/CIRCRESAHA.121.318224 PMC8285054 34110911

[20] Trujillo Rivera EA Chamberlain JM Patel AK Morizono H Heneghan JA Pollack MM. Dynamic Mortality Risk Predictions for Children in Icus: Development and Validation of Machine Learning Models. Pediatr Crit Care Med (2022) 23(5):344–52. doi: 10.1097/PCC.0000000000002910 PMC9117400 35190501

[21] McLaughlin M Pelle KG Scarpino SV Giwa A Mount-Finette E Haidar N. Development and Validation of Manually Modified and Supervised Machine Learning Clinical Assessment Algorithms for Malaria in Nigerian Children. Front Artif Intell (2021) 4:554017. doi: 10.3389/frai.2021.554017 35187469 PMC8851346

[22] Malizia V Cilluffo G Fasola S Ferrante G Landi M Montalbano L. Endotyping Allergic Rhinitis in Children: A Machine Learning Approach. Pediatr Allergy Immunol (2022) 33 Suppl 27:18–21. doi: 10.1111/pai.13620 35080305 PMC9546471

[23] Just AC Liu Y Sorek-Hamer M Rush J Dorman M Chatfield R. Gradient Boosting Machine Learning to Improve Satellite-Derived Column Water Vapor Measurement Error. Atmos Meas Tech (2020) 13(9):4669–81. doi: 10.5194/amt-13-4669-2020 PMC7665162 33193906

[24] Sorensen S Brix N Ernst A Lauridsen LLB Ramlau-Hansen CH. Maternal Age at Menarche and Pubertal Development in Sons and Daughters: A Nationwide Cohort Study. Hum Reprod (2018) 33(11):2043–50. doi: 10.1093/humrep/dey287 30312405

[25] Yang B Ostbye T Huang X Li Y Fang B Wang H. Maternal Age at Menarche and Pubertal Timing in Boys and Girls: A Cohort Study From Chongqing, China. J Adolesc Health (2021) 68(3):508–16. doi: 10.1016/j.jadohealth.2020.06.036 32798100

[26] Bacopoulou F Efthymiou V Landis G Rentoumis A Chrousos GP. Waist Circumference, Waist-To-Hip Ratio and Waist-To-Height Ratio Reference Percentiles for Abdominal Obesity Among Greek Adolescents. BMC Pediatr (2015) 15:50. doi: 10.1186/s12887-015-0366-z 25935716 PMC4446827

[27] Wang T Ma X Tang T Jin L Peng D Zhang R. Overall and Central Obesity With Insulin Sensitivity and Secretion in a Han Chinese Population: A Mendelian Randomization Analysis. Int J Obes (Lond) (2016) 40(11):1736–41. doi: 10.1038/ijo.2016.155 27569682

[28] Roemmich JN Rogol AD. Role of Leptin During Childhood Growth and Development. Endocrinol Metab Clin North Am (1999) 28(4):749–64. doi: 10.1016/s0889-8529(05)70100-6 10609118

[29] Liu G Guo J Zhang X Lu Y Miao J Xue H. Obesity Is a Risk Factor for Central Precocious Puberty: A Case-Control Study. BMC Pediatr (2021) 21(1):509. doi: 10.1186/s12887-021-02936-1 34784914 PMC8594221

[30] Calthorpe L Brage S Ong KK. Systematic Review and Meta-Analysis of the Association Between Childhood Physical Activity and Age at Menarche. Acta Paediatr (2019) 108(6):1008–15. doi: 10.1111/apa.14711 PMC6563453 30588652

[31] Shokri E Heidarianpour A Razavi Z. Positive Effect of Combined Exercise on Adipokines Levels and Pubertal Signs in Overweight and Obese Girls With Central Precocious Puberty. Lipids Health Dis (2021) 20(1):152. doi: 10.1186/s12944-021-01588-5 34742317 PMC8571828

[32] Mihrshahi S Drayton BA Bauman AE Hardy LL. Associations Between Childhood Overweight, Obesity, Abdominal Obesity and Obesogenic Behaviors and Practices in Australian Homes. BMC Public Health (2017) 18(1):44. doi: 10.1186/s12889-017-4595-y 28732475 PMC5521098

[33] Perez-Farinos N Villar-Villalba C Lopez Sobaler AM Dal Re Saavedra MA Aparicio A Santos Sanz S. The Relationship Between Hours of Sleep, Screen Time and Frequency of Food and Drink Consumption in Spain in the 2011 and 2013 Aladino: A Cross-Sectional Study. BMC Public Health (2017) 17(1):33. doi: 10.1186/s12889-016-3962-4 28056890 PMC5217644

[34] Stagi S De Masi S Bencini E Losi S Paci S Parpagnoli M. Increased Incidence of Precocious and Accelerated Puberty in Females During and After the Italian Lockdown for the Coronavirus 2019 (Covid-19) Pandemic. Ital J Pediatr (2020) 46(1):165. doi: 10.1186/s13052-020-00931-3 33148304 PMC7609833

[35] Henderson M Gray-Donald K Mathieu ME Barnett TA Hanley JA O'Loughlin J. How Are Physical Activity, Fitness, and Sedentary Behavior Associated With Insulin Sensitivity in Children? Diabetes Care (2012) 35(6):1272–8. doi: 10.2337/dc11-1785 PMC3357250 22492585

